# Comparing the Ecological Stoichiometry in Green and Brown Food Webs – A Review and Meta-analysis of Freshwater Food Webs

**DOI:** 10.3389/fmicb.2017.01184

**Published:** 2017-06-29

**Authors:** Michelle A. Evans-White, Halvor M. Halvorson

**Affiliations:** ^1^Department of Biological Sciences, University of Arkansas, FayettevilleAR, United States; ^2^Department of Biological Sciences, University of Southern Mississippi, HattiesburgMS, United States

**Keywords:** food quality, detrital food webs, light nutrient hypothesis, growth, excretion, egestion

## Abstract

The framework of ecological stoichiometry was developed primarily within the context of “green” autotroph-based food webs. While stoichiometric principles also apply in “brown” detritus-based systems, these systems have been historically understudied and differ from green ones in several important aspects including carbon (C) quality and the nutrient [nitrogen (N) and phosphorus (P)] contents of food resources for consumers. In this paper, we review work over the last decade that has advanced the application of ecological stoichiometry from green to brown food webs, focusing on freshwater ecosystems. We first review three focal areas where green and brown food webs differ: (1) bottom–up controls by light and nutrient availability, (2) stoichiometric constraints on consumer growth and nutritional regulation, and (3) patterns in consumer-driven nutrient dynamics. Our review highlights the need for further study of how light and nutrient availability affect autotroph–heterotroph interactions on detritus and the subsequent effects on consumer feeding and growth. To complement this conceptual review, we formally quantified differences in stoichiometric principles between green and brown food webs using a meta-analysis across feeding studies of freshwater benthic invertebrates. From 257 datasets collated across 46 publications and several unpublished studies, we compared effect sizes (Pearson’s r) of resource N:C and P:C on growth, consumption, excretion, and egestion between herbivorous and detritivorous consumers. The meta-analysis revealed that both herbivore and detritivore growth are limited by resource N:C and P:C contents, but effect sizes only among detritivores were significantly above zero. Consumption effect sizes were negative among herbivores but positive for detritivores in the case of both N:C and P:C, indicating distinct compensatory feeding responses across resource stoichiometry gradients. Herbivore P excretion rates responded significantly positively to resource P:C, whereas detritivore N and P excretion did not respond; detritivore N and P egestion responded positively to resource N:C and P:C, respectively. Our meta-analysis highlights resource N and P contents as broadly limiting in brown and green benthic food webs, but indicates contrasting mechanisms of limitation owing to differing consumer regulation. We suggest that green and brown food webs share fundamental stoichiometric principles, while identifying specific differences toward applying ecological stoichiometry across ecosystems.

## Introduction

Ecological stoichiometry was developed and has been considered extensively within the context of autotroph-based, or “green” food webs (Sterner and Elser, 2002) that conform nicely to the classic trophic level concept of primary producers and upper level consumers (Lindeman, 1942; Hairston et al., 1960). Although most energy and organic nutrients available to organisms are ultimately derived from autotrophs, the majority of energy [carbon (C)] fixed by primary producers enters the pool of detritus and becomes part of the “brown,” detritus-based food web (Cebrian, 1999; Cebrian and Lartigue, 2004). Brown food webs remain comparatively under-studied by ecological stoichiometry theory, but the framework can provide insight into controls on brown trophic processes by examining the interplay of materials and energy between detritus, decomposer microbes, and detritivores (Moore et al., 2004). While there are shared stoichiometric constraints, there are still notable differences between green and brown food webs. For example, unlike green food webs in which herbivores directly ingest but do not themselves contribute organic C and organic nutrients to the autotroph pool, detrital organic carbon and nutrients are repackaged and consumed several times in brown food webs, resulting in a “microbial loop" or “detrital processing chain.” This and other inherent differences may result in distinct stoichiometric principles throughout green versus brown food webs.

In this paper, we use a conceptual review and quantitative meta-analysis to summarize work over the last decade that has developed the application of ecological stoichiometry from green to brown food webs. We first identify three main areas where green and brown food webs differ, yet stoichiometric principles are shared and translate from green to brown systems. First, light is not a direct nutritional resource for heterotrophic organisms and the light and nutrient resource gradient that is recognized as an important control on the autotrophic community and primary production (Sterner et al., 1997) has received less attention within the context of the ecological stoichiometry of brown food webs than green ones. Second, detritivores have evolved with lower quality [<nutrient:C] food resources than herbivores (Frost et al., 2006) and their physiological responses to food resource enrichment may differ, having consequences for community structure and consumer-driven nutrient dynamics (CND). Third, the stoichiometry of CND in brown food webs has received much less consideration than that in green food webs (Moe et al., 2005; Halvorson et al., 2015a; Atkinson et al., 2016), and we highlight how CND may differ between the two trophic systems. We complement our review with a meta-analysis of existing studies from aquatic ecosystems, assessing how stoichiometric constraints on consumer growth, consumption, and waste production (egestion/excretion) compare between green and brown benthic food webs. The meta-analysis provides a focused, quantitative test of several predictions generated by our conceptual review. Throughout this paper we focus on plant litter as the basis of brown food webs, because it is a widespread form of detritus across inland ecosystem types.

## Literature Review – Comparing Ecological Stoichiometry of Green and Brown Food Webs in Three Main Areas

### Comparing Light and Nutrient Effects on the Resource Base of Green and Brown Food Webs

Autotroph stoichiometry varies widely across resource gradients (e.g., light and nutrients) due to their ability to store nutrients beyond what is needed for growth (Sterner et al., 1997; Persson et al., 2010). Autotrophs also tend to have lower N:C and P:C ratios than heterotrophs due to the presence of a cell wall and greater structural C material like cellulose and lignin (Sterner and Elser, 2002). Algal N:C and P:C tend to be lower than terrestrial plant tissue due to the presence of more structural material in plants (Elser et al., 2000), and the stoichiometry of different tissues varies across leaves, stems, wood, and roots (Sterner and Elser, 2002). Given this variation, plant litter that contributes regularly to detrital pools varies widely across species and biomes (Mcgroddy et al., 2004; Cornwell et al., 2008; Vergutz et al., 2012). Large particulate detritus tends to have even lower N:C and P:C than living plant tissue, due to resorption and leaching of soluble compounds after senescence. This resorption and leaching results in generally N- and P-deplete resources at the base of brown compared to green food webs (Lemoine et al., 2014).

One complication in examining basal food resource stoichiometry for macroconsumers in brown relative to green food webs is that detrital stoichiometry is derived from autotrophic as well as microbial heterotrophic decomposer tissue. Heterotrophic bacteria and fungi tend to have higher N:C and P:C ratios than leaf litter (Makino et al., 2003; Danger and Chauvet, 2013) and elemental imbalances between the microbial decomposers and the detritus can be alleviated at the organismal level by flexible stoichiometry or by changing physiological efficiencies (Manzoni et al., 2008, 2010; Kaiser et al., 2014; Manzoni, 2017). The limited data available for fungi suggest that they can have flexible nutrient:C ratios across resource gradients and that their biomass can range more broadly in elemental composition than other heterotrophs (Danger and Chauvet, 2013; Danger et al., 2016); however, autotroph elemental composition still varies more broadly than heterotrophs (Sterner and Elser, 2002; Mcgroddy et al., 2004). Bacteria can also have variable P:C stoichiometry across strains (Scott et al., 2012) and some are more homeostatic than others (Cotner et al., 2006, 2010; Godwin and Cotner, 2014, 2015). Together, this variation across heterotrophic microbes results in a greater possible range of detrital stoichiometry, additional to that attributable to variation across plant tissues alone (Fanin et al., 2013).

Nutrients and light availability are key controls on resources in both green and brown food webs, because both factors can stimulate primary production, increasing the flux and changing the chemical quality of autotroph material that interacts with or enters the detrital pool (Gusewell and Gessner, 2009; Valera-Burgos et al., 2013; Liu et al., 2016). Nutrient enrichment often alleviates autotroph growth limitation, enhancing biomass (Elser et al., 2007) and increases the N:C and P:C of algal tissue and the nutrient:lignin and nutrient:C ratios of plants (Coulson and Butterfield, 1978; Aerts, 1997; Xu and Hirata, 2005). Further, nutrients may interact with light availability to determine the stoichiometry of autotrophs; the nutrient:light hypothesis (note we have switched numerator and denominator to provide consistency with our use of nutrient:C ratios) suggests that the balance between these two autotroph resources regulates autotroph nutrient:C ratios. Autotroph nutrient:C ratios should be positively related to the nutrient:light ratio (Sterner et al., 1997). Tests of the nutrient:light hypothesis have primarily focused on pelagic ecosystems and relationships between P:light and seston or autotroph P:C (Sterner et al., 1997). A few studies have applied the nutrient:light hypothesis to benthic aquatic algae (Hill and Fanta, 2008; Hill et al., 2009; Fanta et al., 2010), but few studies have extended it to terrestrial plants and they have primarily focused on plant:mycorrhizal interactions (Elliott and White, 1994; Treseder, 2004; Johnson et al., 2010). This extension will be key to understanding the broader applicability of this stoichiometric concept across interfaces of green and brown trophic systems, especially because terrestrial plant litter provides a major resource base for brown food webs.

As in green food webs, nutrient enrichment in brown food webs increases the P:C and N:C ratios of basal food resources because microbial decomposers on detritus are capable of assimilating dissolved N and P from the water column (Suberkropp and Chauvet, 1995; Cheever et al., 2013; Scott et al., 2013). Since heterotrophic bacteria and fungi can have weakly flexible N:C and P:C that are higher than the detrital substrate (Makino et al., 2003; Danger and Chauvet, 2013), their growth and nutrient storage can result in increased N and P contents of detritus during decomposition. Notably, increased microbial biomass also enhances the quality of detrital C, through accumulation of microbial lipids, soluble carbohydrates, and protein that are nutritionally valuable compared to plant polysaccharides like cellulose and lignin that dominate detrital substrate C and are resistant to breakdown and assimilation (Martin et al., 1980; Chung and Suberkropp, 2009a,b). As elevated nutrients stimulate microbial growth, increased decomposition rates often accompany nutrient enrichment (Ferreira et al., 2015; Kominoski et al., 2015; Manning et al., 2015, 2016), stimulating C loss from ecosystems (Benstead et al., 2009; Rosemond et al., 2015). In this way, nutrient enrichment increases the quality (nutrient:C) of basal food resources in both green and brown food webs. However, enrichment has contrasting effects on resource quantity because nutrients stimulate autotroph growth, enhancing resource quantity in green food webs, whereas nutrients increase decomposition rates and therefore reduce resource quantity in brown food webs (Rosemond et al., 2015).

The role of light availability in brown food webs is less clear than in green food webs, because microbial decomposers cannot directly use light as a resource and detritus is only affected directly by light through photolysis that stimulates breakdown (Wetzel et al., 1995). The role of light in decomposition has been largely neglected under the assumption that most decomposition occurs in low-light environments with minimal algal biomass (Fisher and Likens, 1973). However, sufficient light can occur in many aquatic settings, where light permits algal growth on detritus, changing the microbial assemblage and altering detrital stoichiometry and decomposition (Lagrue et al., 2011; Danger et al., 2013a; Kuehn et al., 2014). Notably, periphytic algae could reduce detrital P:C or N:C under low nutrient levels, but increase the maximum detrital P:C or N:C under high nutrient levels, because of autotrophs’ greater stoichiometric flexibility and ability to store excess nutrients (Persson et al., 2010; Danger et al., 2013a; Halvorson et al., 2016a). A key indirect effect of light may also be to “prime” decomposition because algae exude fresh, labile C that may be used by fungi and bacteria to invest in growth or enzyme production, stimulating breakdown of recalcitrant detritus via the priming effect (Kuzyakov et al., 2000; Guenet et al., 2010; Kuehn et al., 2014). This coupling of periphytic autotrophs and heterotrophs may depend on the nutrient:light ratio, which influences autotroph nutrient:C ratios and algal C exudation rates that probably elicit the priming effect (Sterner et al., 1997; Guenet et al., 2010; Wyatt and Turetsky, 2015). Existing studies suggest high light and nutrient levels suppress decomposition through negative priming effects, whereas high light and low nutrient levels stimulate decomposition through positive priming effects (Danger et al., 2013a; Halvorson et al., 2016a). Further studies are clearly needed to address the interactive effects of light and nutrients on brown food webs, especially regarding variation in detrital stoichiometry and recalcitrance, the stoichiometry of algal-heterotroph interactions, and implications of periphytic algae for detrital food quality to detritivores (Guo et al., 2016).

### Effects of Food Resource Nutrient Enrichment in Brown and Green Food Webs

Physiological responses to food resource elemental ratios are central to understanding organismal homeostasis, growth, fitness, and nutrient cycling (Sterner and Elser, 2002; Cross et al., 2005; Frost et al., 2005a; Sperfeld et al., 2017). Although the degree of stoichiometric homeostasis varies across metazoans (Persson et al., 2010), often some degree of constraint on body elemental contents and ratios occurs due to biomolecular composition, body plans, and life history traits (Elser et al., 1996; Sterner and Elser, 2002). These constraints are common and shape stoichiometric principles throughout a diversity of food webs. Indeed, since the turn of the century, studies have shown herbivores and detritivores often have higher N:C and P:C ratios than their food resources (Elser et al., 2000; Lemoine et al., 2014) potentially leading to widespread nutrient limitation of growth. Understanding how resource stoichiometry affects consumer growth and physiology is key to comparing stoichiometric constraints in green and brown food webs, including under anthropogenic enrichment that broadly increases resource N:C and P:C (Cross et al., 2003; Peñuelas et al., 2013).

Herbivore and detritivore responses to nutrient enrichment will likely differ due to contrasting stoichiometry and C quality (recalcitrance and digestibility) of autotroph versus detrital food resources. Because organism nutrient:C ratios often decrease as one moves from unicellular autotrophs to land plants, terrestrial herbivores and detritivores, as well as aquatic detritivores, rely on food resources of lower nutrient contents compared to aquatic herbivores (Cebrian and Lartigue, 2004). These taxa have therefore likely faced greater elemental imbalances during their evolutionary history than aquatic herbivores, and may have evolved lower demands for nutrients in food resources (Frost et al., 2006). As autotroph nutrient:C ratios decrease, C quality also declines across the spectrum from unicellular autotrophs to vascular plants, leading to greater digestion resistance and constraining the proportion of resource C assimilated by consumers of vascular plant tissue (Sterner and Elser, 2002; Cebrian, 2004). The quality of C available further differs between living, actively growing plant material consumed by herbivores versus dead plant litter consumed by detritivores (Vergutz et al., 2012), setting an additional contrast between resources of the two trophic groups. These differences will shape consumers’ response to nutrient enrichment because C assimilation constrains animals’ ability to use ingested nutrients (DeMott et al., 2010; DeMott and Van Donk, 2013). Together, these trends support a general prediction that herbivores may be better-equipped to respond positively to resource nutrient enrichment, relative to detritivores.

The consumption response to resource stoichiometry is an important component of growth, but the direction (positive or negative) and magnitude in response to nutrient enrichment may differ between herbivores and detritivores. Detritivores targeting the acquisition of limiting resources may increase their consumption rates (Ott et al., 2012; Flores et al., 2014; Fuller et al., 2015) or selectively feed on food resources more rich in potentially limiting nutrients (Frainer et al., 2016). On the other hand, herbivores tend to exhibit reduced consumption on higher-nutrient diets (Plath and Boersma, 2001; Boersma and Elser, 2006; Fink and Von Elert, 2006) and detritivore consumption rates increase at a similar rate with the nutrient content and the production of their autotrophic and detrital food resources across terrestrial and aquatic ecosystems (Cebrian and Lartigue, 2004). Therefore, we may expect aquatic herbivores and detritivore consumption rates to increase similarly as their food resources become enriched although herbivore responses may be weaker than detritivores’.

The complexity of detritivore food resources (e.g., recalcitrant N bound to lignin; Chapin et al., 2002) and lower nutrient content (Cross et al., 2003) relative to living autotrophic tissue may result in lower detritivore assimilation efficiencies (AEs) and lower GGEs compared to herbivores. A meta-analysis found that detritivores tended to have a lower C GGE than herbivores (Frost et al., 2006), but other element-specific GGEs and AEs were not commonly available across feeding guilds, and it remains unclear how efficiently detritivores assimilate and convert nutrients into new growth. However, recent estimates for aquatic detritivore element-specific AEs and GGEs (Halvorson et al., 2015b, 2016b) suggest that N- and P-specific AE and GGE are lower than those estimated for aquatic herbivores (DeMott et al., 1998; Ferrão-Filho et al., 2007). Therefore, detritivores will likely excrete elements at lower and egest at higher rates than taxa in other feeding guilds (McManamay et al., 2011). This trend is likely to persist even with nutrient enrichment of food resources, because nutrient enrichment does not appear to improve detritivore AE or GGE, possibly because the recalcitrance of detrital C ultimately constrains detritivores’ ability to invest energy or resources toward acquisition of added nutrients (Halvorson et al., 2015b, 2016b).

Bioenergetic models indicate that aquatic herbivores have a greater growth demand for P relative to C (i.e., higher P:C threshold elemental ratios) and greater C GGEs than do aquatic detritivores (Frost et al., 2006). A positive relationship between P demand and growth has been observed across broad taxa (Elser et al., 2003) and across aquatic taxa (Frost et al., 2006; Benstead et al., 2014) suggesting aquatic detritivores may have traded the ability to grow fast for the ability to utilize food resources with a low P:C (i.e., terrestrial detritus). Even within herbivorous zooplankton, species C- and P- specific growth rates are coupled and growth rate is an important predictor of taxa responses to P enrichment of food resources (Hood and Sterner, 2014). Aquatic herbivores may have evolved greater growth rates and may have a greater capacity for growth responses to nutrient enrichment of food resources compared to aquatic detritivores. We address many of these questions below in a meta-analysis comparing aquatic herbivore and detritivore responses to resource N:C and P:C in controlled feeding studies.

### Comparing Consumer-Driven Nutrient Dynamics in Green and Brown Food Webs

Consumers can play important roles in ecosystem nutrient dynamics (Elser and Urabe, 1999; Vanni, 2002; Pastor et al., 2006), but these roles likely differ in green and brown food webs due to contrasting resource stoichiometry and recalcitrance, as well as differing processing of consumer wastes after release. Most studies of consumer-driven nutrient dynamics (CND) have focused on herbivores in pelagic green food webs, where the unidirectional flow of energy and nutrients and tight consumer-resource feedbacks may, in part, simplify CND (Elser and Urabe, 1999). While CND can be easily translated across systems, CND is probably more complex in terrestrial systems and in aquatic brown food webs, because multiple forms of waste – including excreta, egesta, exuvia, and carcasses – must be considered as components of CND, with potential to affect nutrient availability and consumer-resource feedbacks (Vanni et al., 2013; Sitters et al., 2017). In particular, the iterative re-packaging and processing of detritus along a transfer chain may result in multiple steps and controls on the strength of CND in brown food webs (Heal and Maclean, 1975; Navel et al., 2011; Bundschuh and Mckie, 2016). Further understanding of CND in brown and green food webs will be important to quantify the broad roles of animals in ecosystem nutrient cycles (Vanni, 2002; Atkinson et al., 2016), including under prevalent “multichannel” feeding by omnivorous taxa (Wolkovich et al., 2014).

Ecological stoichiometry has historically focused on dissolved and bioavailable excreta rather than particulate wastes like egesta, because autotrophs are capable of directly assimilating excreta, forming direct consumer-resource nutrient feedbacks (Sterner, 1986; McNaughton et al., 1997b; Elser and Urabe, 1999; Evans-White and Lamberti, 2005). Moreover, dissolved excreta are often considered the dominant nutrient waste flux from the consumer pool (Zanotto et al., 1993; DeMott et al., 1998); these assumptions are directly tied to the natural history and community structure of green food webs (but see Higgins et al., 2006). However, brown food webs can also show a tight interplay between consumer wastes and heterotrophic activity, because microbial heterotrophs are capable of assimilating consumer excreta (Fornara and Du Toit, 2008; Cheever et al., 2012; Rugenski et al., 2012; Villanueva et al., 2012). In this way, consumer nutrient recycling is likely to promote biomass turnover of both autotrophic and heterotrophic microbes (Hill and Griffiths, 2017); however, in green food webs with plentiful light, this may come with minimal reductions in autotroph standing stocks (Hobbs, 1996; Knoll et al., 2009), whereas in brown food webs with limited detrital stocks, consumers will enhance decomposition both directly via consumption and indirectly via nutrient recycling that stimulates heterotrophy.

Studies from both green and brown food webs increasingly consider nutrient wastes released as egesta (Liess and Haglund, 2007; Hood et al., 2014; Halvorson et al., 2015a). Nutrient egestion rates by aquatic herbivores and detritivores can equal or exceed excretion rates (Hood et al., 2014; Liess, 2014; Halvorson et al., 2015a; Norlin et al., 2016). In terrestrial settings, both egesta and excreta are substantial, often concurrent nutrient subsidies of consumers to soils (McNaughton et al., 1997a; Clay et al., 2014; Sitters et al., 2014), and both forms of waste have historically been considered as important pathways of CND (Hobbs, 1996). The relative importance of egestion versus excretion as components of CND will likely vary with the resource N and P contents (Zanotto et al., 1993; Hobbs, 1996; Halvorson et al., 2015a) and the recalcitrance of ingested nutrients, including whether ingested nutrients are bound in living versus dead tissues. The recalcitrance of associated C may also set limits on assimilation and subsequent growth and storage of nutrients in animal tissues (Atkinson et al., 2016). Given greater recalcitrance of detrital C and nutrients compared to autotrophic C and nutrients, egestion is likely to play a relatively greater role in CND in brown food webs than in green food webs. However, the ecological importance of egestion versus excretion will also depend on environmental processing of each form of waste (Liess and Haglund, 2007; Sperfeld et al., 2016); egesta, in particular, can play diverse roles in nutrient dynamics because they are subject to microbial breakdown, direct ingestion by animals, and transport/deposition (Wotton and Malmqvist, 2001). Egested nutrients probably occur in recalcitrant forms that limit the rate and magnitude of nutrient release, slowing nutrient turnover relative to excretion (Liess and Haglund, 2007; Sperfeld et al., 2016). Furthermore, decomposing egesta may exhibit uptake of inorganic nutrients to support microbial growth, which would slow ecosystem-level nutrient turnover (Halvorson et al., 2017). As a subsidy of C and nutrients to depositional zones like soil or the aquatic hyporheos, egestion probably fuels ecosystem respiration and supports the subterranean food web (Navel et al., 2011). Overall, the fates of animal egesta versus excreta must be further studied to holistically understand CND, especially in brown food webs (Navel et al., 2011; Bundschuh and Mckie, 2016).

The lower nutrient content of detrital resources, compared to living plant matter, may indicate brown food webs to be more strongly nutrient-limited than green food webs, and therefore animals may be generally less-efficient recyclers of nutrients in brown food webs. This is consistent with evidence that aquatic detritivorous animals display lower N and P excretion rates than their herbivorous counterparts (McManamay et al., 2011), but comparisons from additional settings are clearly needed. Moreover, generalizations of bulk detritus as the stoichiometry of ingested resources are likely to underestimate excretion and egestion rates (Hood et al., 2014). This is because detritivorous animals selectively feed on nutrient-rich biofilms on detritus. Such selective feeding likely varies across animal species (Arsuffi and Suberkropp, 1989) and confounds predictions of aquatic CND across animals (Dodds et al., 2014). Predictions of CND could be aided by quantifying the degree of selectivity across species and identifying trends across coarse traits such as mouthpart morphology, trophic mode, or body size (Dodds et al., 2014), as done among large terrestrial herbivores (Pastor et al., 2006). This work is necessary to accurately place animals within ecosystem processes, including consumption, release, and storage of nutrients, and thereby understand how CND may depend on an ecosystem’s trophic basis (Atkinson et al., 2016; Hill and Griffiths, 2017).

In many systems, CND may also provide a link between seemingly disparate nutrient dynamics in green and brown food webs (Cherif and Loreau, 2013; Zou et al., 2016). Because autotrophs and heterotrophs share the same pool of inorganic nutrients, inorganic wastes from consumers can easily interchange between detritus and autotrophs, resulting in complex interplay between trophic processes in each food web (Zou et al., 2016). Moreover, herbivores themselves produce organic wastes including egesta, and these wastes are subject to microbial and other breakdown processes within the pool of detritus, but do not return to autotrophs until mineralization (Hawlena and Schmitz, 2010). The entanglement of CND between green and brown food webs challenges the traditional dichotomy between these energy flow channels, leading toward weaker consumer-resource nutrient feedbacks when a consumer’s nutrient wastes are incorporated by a food resource inaccessible to that consumer (i.e., herbivore excreta are assimilated by heterotrophic decomposers; Fornara and Du Toit, 2008; Zou et al., 2016). The nutrient interchange between green and brown food webs also occurs when omnivores consume and subsequently recycle nutrients derived from both autotrophs and detritus (Polis and Strong, 1996; Wolkovich et al., 2014). In this way, CND provides a connection between green and brown food webs, but may not facilitate the tight feedbacks between consumers and their resources originally conceived by ecological stoichiometry theory (Elser and Urabe, 1999).

## Meta-Analysis of Freshwater Benthic Invertebrate Feeding Studies to Quantitatively Compare Ecological Stoichiometry in Brown and Green Food Webs

### Methods

We sought to assess the current literature regarding stoichiometric constraints on organismal growth and stoichiometric regulation in green and brown food webs, because many existing studies remain limited to single or a handful of similar taxa, and there has been little synthesis across the breadth of studies, and few formal comparisons between green and brown food webs (but see Lemoine et al., 2014). We collected data on freshwater benthic invertebrate herbivore and detritivore taxa that had been fed food resources where nutrient:C ratios were controlled or manipulated. Published datasets were identified using the following search strings in Web of Science, searched on September 15, 2016 (TS means “topic search”; keywords): TS = (herbivor^∗^ OR graz^∗^ OR detritivor^∗^ OR invertebrate OR shredd^∗^ OR macroinvertebrate OR zooplankton) AND TS = (stoichiometr^∗^). This search yielded 1,144 studies, from which we identified publications suitable for data extraction. Although we initially planned to include zooplankton, we narrowed our selection to benthic invertebrates to focus the meta-analysis. We supplemented the Web of Science search with a Google Scholar search of 2,000 additional hits for more recent literature and dissertations/theses (excluding any duplicate publications). From each study, we used figures (extraction using DataThief), tables, and appendices to collect the following variables where available: diet N:C and P:C, growth rates, consumption rates, and N and P excretion and egestion rates. We also noted sample sizes, consumer trophic mode (detritivore or herbivore), consumer and diet taxonomy, whether dietary gradients were monospecific or across multiple species, and temperature. To a total of 46 published studies ultimately included in the meta-analysis, we added eight unpublished studies of our own. Because many publications reported data from >2 experiments such as at multiple temperatures, contrasting diet types (e.g., litter or algal species) or from multiple consumer species, we treated each experiment as an independent dataset suitable for inclusion in the meta-analysis. Note our meta-analysis assumed independence of datasets among closely-related taxa and when datasets were from the same study or research group. Where studies used only two levels of resource N:C or P:C, we obtained raw data from the corresponding author to permit calculation of effect size. We also excluded datasets in which minimum and maximum mean resource N:C or P:C overlapped within 1 SD, ensuring a robust gradient of resource stoichiometry (Halvorson and Small, 2016). Altogether, 257 datasets were included in the meta-analysis.

From each dataset, we calculated effect sizes of resource P:C or N:C (Pearson’s r) on each response variable (growth, consumption, excretion, or egestion), such that positive effects indicate a positive response to food resource nutrient enrichment (Persson et al., 2010). Pearson’s r was transformed to Fisher’s Z and weighted according to its variance as [1/(*n*-3)] where *n* = sample size for a dataset (Rosenberg et al., 2013). We used a weighted mixed effects model to test differences in effect size between detritivorous and herbivorous taxa (Rosenberg, 2013). This model treated trophic mode (categories = herbivore or detritivore) as a fixed effect and dataset identity as a random effect. The use of random effects accounts for heterogeneity across studies due to variable factors including temperature, taxonomy, and diet. We assessed heterogeneity of effect sizes across studies using the I^2^ statistic, which equates to the proportion of total heterogeneity attributable to between-study variance (**Table 1**) (Senior et al., 2016). Because of insufficient datasets regarding N and P egestion by herbivores, we decided to exclude herbivores from the meta-analysis of those effect sizes and focus only on detritivore datasets. I^2^ and the random variance terms are calculated only for a global mean model (null hypothesis = effect size of zero) in those sets, accordingly. We also used one-sample weighted *t*-tests to determine if effect sizes differed from a null hypothesis of *Z* = 0 (no response to resource stoichiometry) for each trophic mode in each analysis. All statistics were conducted using R version 3.3.1 (R Core Team, 2013) and the R package ‘weights’ (Pasek, 2016).

**Table 1:** Sample sizes, I^2^, and random effects variance for each of eight variables in response to resource N:C or P:C manipulations in the met-analysis.

Response variable	Resource manipulation	# Datasets	I^2^	Random effects variance
Growth	N:C	39	93.6%	0.486
Growth	P:C	54	96.5%	0.900
Consumption	N:C	44	96.2%	0.638
Consumption	P:C	34	96.6%	0.572
N excretion	N:C	15	87.5%	0.423
P excretion	P:C	23	96.5%	1.221
N egestion	N:C	21	92.8%	0.730
P egestion	P:C	27	91.0%	0.584

*For a summary of all datasets and effect sizes, see Supplementary Table 1.*

### Results

We report sample sizes, I^2^, and random effects variance of the meta-analysis in **Table 1**. Further description of datasets and associated effect sizes and citations can be found in Supplementary Table 1.

**FIGURE 1:**
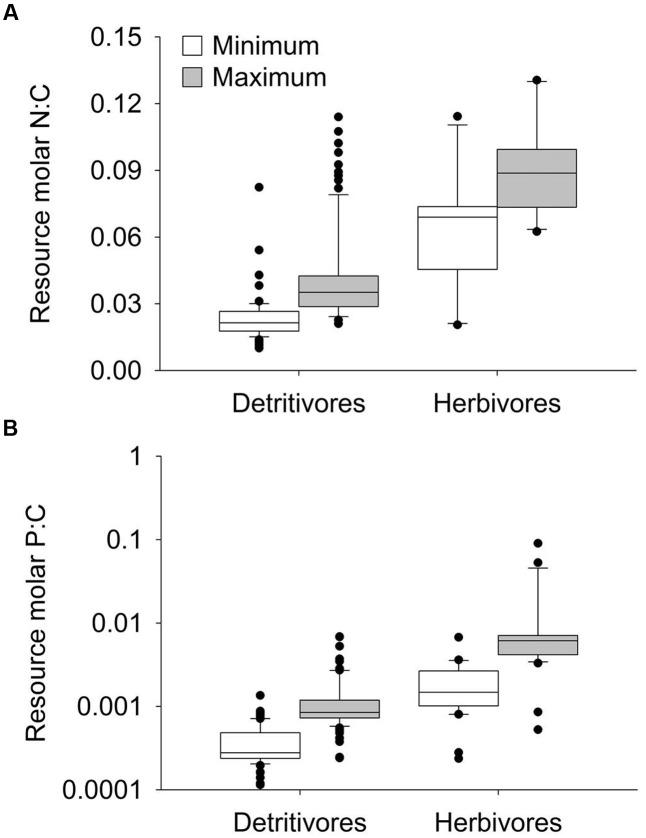
Boxplot of minimum and maximum resource N:C contents **(A)** and P:C contents **(B)** in benthic detritivore and herbivore feeding studies included in the meta-analysis. Note the logarithmic Y-axis in **(B)**. The black horizontal lines indicate median values within each group.

Across feeding studies included in the meta-analysis, resource N:C and P:C contents spanned a wide range across all datasets (**Figure 1**). Although there was notable overlap in the overall range, herbivores’ resources (autotrophs) were generally greater in N:C and P:C contents compared to detritivores’ resources (detritus; **Figure 1**). The datasets spanned organisms from eight taxonomic orders, with most herbivore studies using Gastropoda and detritivore studies showing a broader diversity, but primarily using Trichoptera, Plecoptera, Amphipoda, and Diptera (Supplementary Figure 1). Most studies used organisms from streams or rivers, followed by lakes and wetlands/ponds (Supplementary Figure 2).

**FIGURE 2:**
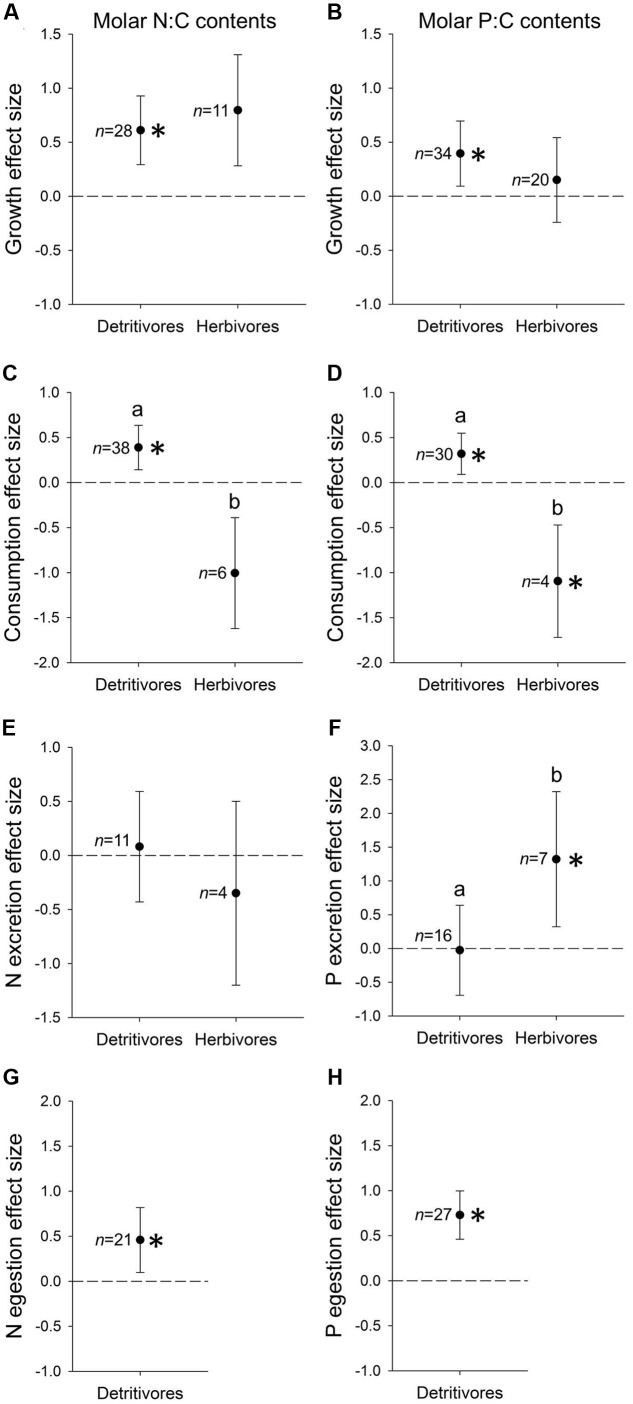
Mean ± 95% CI effect sizes (weighted Z-scores) of resource molar N:C contents **(A,C,E,G)** and P:C contents **(B,D,F,H)** on rates of growth **(A,B)**, consumption **(C,D)**, N excretion **(E)**, P excretion **(F)**, N egestion **(G)**, and P egestion **(H)** of detritivorous and herbivorous benthic invertebrates. Sample sizes (*n*) are indicated to the left of each effect size. Asterisks indicate effect sizes different from zero (*t*-test, *P* < 0.05). Letters indicate effect sizes differ between trophic modes (ANOVA, *P* < 0.05). Only detritivores were included in **(G,H)** due to insufficient egestion datasets from herbivores (*n* = 2).

Detritivore and herbivore growth responses to resource N:C contents were similar and positive, although only the detritivore response was significantly greater than zero (*t*_1,27_ = 5.39, *P* < 0.001; **Figure 2A**). The two trophic modes also did not differ in growth responses to resource P:C. Detritivorous taxa showed a positive P:C-growth response significantly greater than zero (*t*_1,33_ = 3.14; *P* < 0.01) whereas the herbivore response did not differ from zero (**Figure 2B**).

Effect sizes of resource N:C on herbivore consumption were significantly lower than effects on detritivore consumption (*P* < 0.001; **Figure 2C**). Herbivore consumption responded negatively to resource N:C, but mean effect size was not different from zero, whereas detritivore consumption rates responded significantly positively to resource N:C (*t*_1,37_ = 3.31, *P* < 0.01; **Figure 2C**). Similarly, herbivores and detritivores differed significantly in the effect size of P:C on consumption (*P* < 0.001; **Figure 2D**). The P:C-consumption effect size was significantly greater than zero for detritivores (*t*_1,29_ = 2.75, *P* < 0.05) whereas that of herbivores was below zero (*t*_1,3_ = 3.22; *P* < 0.05; **Figure 2D**).

The effects of resource N:C on N excretion did not differ between trophic modes, and neither mode exhibited effect sizes significantly different from zero (**Figure 2E**). In contrast, the effect size of P:C on P excretion differed between trophic modes, with herbivores displaying a higher, positive effect size significantly greater than zero (*t*_1,6_ = 3.63, *P* < 0.05) compared to an effect size indistinguishable from zero among detritivores (**Figure 2F**).

We limited our meta-analysis of N and P egestion to detritivores because we obtained only two herbivore datasets. The response of detritivore N egestion to resource N:C was significantly greater than zero (*t*_1,20_ = 2.49, *P* < 0.02; **Figure 2G**), as was the response of P egestion to resource P:C (*t*_1,26_ = 5.31, *P* < 0.001; **Figure 2H**).

### Discussion

Our meta-analysis of feeding studies supports broad N and P growth limitation among both herbivorous and detritivorous freshwater invertebrates. However, only detritivores exhibited N and P growth effect sizes significantly different from zero (**Figures 2A,B**), suggesting that counter to our predictions in the review above, detritivores’ growth responses to nutrient enrichment may actually be stronger than herbivores’. The greater strength and consistency of limitation among detritivores may be partly attributable to larger sample sizes from detritivorous taxa throughout the meta-analysis, highlighting a literature gap of feeding studies from benthic herbivores, especially among non-Gastropoda (Supplementary Figure 1). Despite limited sample sizes, the mechanisms of growth limitation also appear to differ between trophic modes, given contrasting responses of consumption and P excretion to food resource nutrient enrichment (**Figures 2C,D,F**). These results indicate distinct responses of brown versus green benthic food webs to nutrient enrichment, likely driven by inherent differences in the stoichiometry (**Figure 1**) and C quality of detrital versus autotrophic resources.

Compared to herbivores, detritivores may display stronger and less variable growth responses to elevated resource nutrients because elevated detrital N and P are accompanied by greater C quality in the form of increased microbial biomass (Gulis and Suberkropp, 2003; Manning et al., 2015), whereas autotrophic C quality may only weakly co-vary with N and P contents. Given the importance of microbial C in supporting detritivore growth (Chung and Suberkropp, 2009a; Halvorson et al., 2016b), it is difficult to determine whether positive growth effect sizes are driven by elevated dietary microbial biomass or increased N and P availability. However, one feeding study explicitly manipulated detrital P content without changing fungal biomass and still found strong P-limitation of growth, suggesting P can limit detritivore growth, independent of microbial biomass (Danger et al., 2013b). In the case of autotrophs, increased N and P contents may not affect or may actually drive lower C quality, for example due to diminished eicosapentaenoic acid contents as cyanobacteria form a greater proportion of algal assemblages (Muller-Navarra et al., 2000), which could dampen the herbivore growth response to elevated autrotroph N and P contents. We also note that most herbivore feeding studies (69% of datasets) used resource gradients containing multiple species – especially periphyton composed of multi-species assemblages – which could have weakened or increased variation among herbivore effect sizes. This is in contrast to the majority of detritivore feeding studies (76% of datasets) that employed resource gradients using litter from only one plant species (Supplementary Figure 3). Although microbial taxa on detritus may shift with N or P availability (Lecerf and Chauvet, 2008), a consistent detrital substrate across resource stoichiometry gradients could reduce inter-individual variation and increase growth effect sizes within detritivore feeding studies. While the growth effect sizes are similar, we expect the underlying mechanisms of enhanced growth (e.g., altered consumption or assimilation) to differ between herbivorous and detritivorous taxa, due to inherent contrasts between autotrophic and detrital food resources (see above).

The contrasting consumption effect sizes suggest different bottom–up effects of nutrients on consumption in green versus brown food webs, given benthic detritivores and herbivores exhibit different compensatory feeding with increased resource nutrient content (**Figures 2C,D**). While herbivores may up-regulate consumption on low-nutrient resources, perhaps to increase intake of limiting nutrients (Fink and Von Elert, 2006; Liess, 2014), detritivores up-regulate consumption on high-nutrient resources. This is surprising in light of predictions that both herbivore and detritivore consumption increase positively with resource nutrient enrichment (see review above; Cebrian and Lartigue, 2004). We attribute this dichotomy to the lower nutrient content (**Figure 1**) and low C quality of detritus, relative to that of autotrophs. Detritivores fed low-nutrient resources probably slow their feeding rates to increase gut residence time and maximize assimilation of limiting C and nutrients (Golladay et al., 1983). Indeed, assimilation probably imposes strong limits on detritivore growth, owing to the recalcitrance of detrital C and nutrients that set low maximum assimilation efficiencies (Halvorson et al., 2015b, 2016b). In contrast, herbivores fed low-nutrient resources may retain comparatively high assimilation efficiencies and improve growth by increasing intake rates (Fink and Von Elert, 2006; Liess, 2014). In this way, our meta-analysis suggests herbivores and detritivores exhibit divergent strategies of handling low-nutrient diets and responding positively to nutrient enrichment. Notably, elevated detritivore consumption on high-nutrient litter would contribute to enhanced detritivore-mediated decomposition under nutrient enrichment (Manning et al., 2016), whereas reduced herbivore consumption on high-nutrient diets would alleviate grazing pressure, magnifying the stimulatory bottom–up effects of dissolved nutrients on autotroph biomass (Dodds, 2007).

Although excretion may be an important means for consumers to regulate stoichiometric homeostasis as resources increase in nutrient contents (DeMott et al., 1998; Frost et al., 2005b), we observed no response of N excretion to resource N:C, and only herbivores elevated P excretion on high-P:C resources (**Figures 2E,F**). The small N excretion effect size suggests that in benthic systems, detrital and autotrophic resources may rarely reach a point of excess N contents relative to consumer demands, unlike higher resource P contents that can inhibit growth and are accompanied by elevated P excretion (Boersma and Elser, 2006; Morehouse et al., 2013). One factor shaping these excretion patterns is probably body stoichiometry, especially the stoichiometry of growth, which determines consumer stoichiometric demands (Vanni et al., 2002; Hood and Sterner, 2014; Halvorson et al., 2015b). We did not collect body stoichiometry data in our meta-analysis, but based on limited body stoichiometry data from field-collected benthic invertebrates, body N contents stay consistently high through development and may therefore dampen up-regulated N excretion on high-N:C resources, whereas body P contents often decline during development and could cause individuals to exhibit lower P growth demands and excrete excess P on high-P:C resources (Back and King, 2013). The lack of N or P excretion responses among benthic detritivores suggests other regulatory pathways of nutrient release – namely egestion – may increase when detritivores are fed high-nutrient litter. Indeed, we found detritivores consistently increase N and P egestion when fed high-N:C and high-P:C resources, respectively (**Figures 2G,H**). However, we were unable to assess egestion effect sizes among herbivores, and we reiterate calls for additional excretion and egestion data from diverse taxa, which will help resolve animal nutrient budgets and CND in aquatic ecosystems (McManamay et al., 2011; Vanni and Mcintyre, 2016). One key implication of our meta-analysis is that P enrichment may increase the strength of dissolved CND in green food webs, via increased P excretion, indicative of tight herbivore-autotroph links that we predict in our review. In contrast, brown food webs may exhibit little change in dissolved CND with P enrichment, indicative of weaker detritivore-heterotroph linkages in brown food webs. Instead, nutrient enrichment in brown food webs will elicit strong effects on particulate CND, affecting nutrient availability throughout particle processing chains (Halvorson et al., 2015a).

Our meta-analysis synthesizes current data regarding N and P limitation of freshwater benthic invertebrates, but it carries some weaknesses that limit inferences and should be addressed by future experiments and meta-analyses. First, we narrowed our data collection to controlled feeding studies, primarily from the laboratory, because field studies often have difficulty accurately characterizing resource stoichiometry, face many confounding factors across study sites, and typically have low sample sizes (Halvorson and Small, 2016). During our literature search, however, we found many studies across resource stoichiometry gradients in the field (e.g., Cross et al., 2006; Rothlisberger et al., 2008; McManamay et al., 2011), and a separate meta-analysis of these field studies is warranted to compare effect sizes from controlled studies (see Moody et al., 2015). Second, our meta-analysis addressed consumer limitation by resource N and P separately, but availability of these two elements was likely positively correlated in many studies, and therefore some responses may be driven by increases of N and P together. Among the 46 publications included in our meta-analysis, 27 (59%) manipulated both resource N and P contents. For this reason, we hesitate to explicitly compare effect sizes between the N and P datasets, and we suspect co-limitation by N and P may partly drive the effect sizes in our meta-analysis. Third, we note that herbivore feeding studies on average used higher temperatures (18.2°C) than detritivore feeding studies (10.8°C), which may partly drive different responses between trophic modes (Supplementary Figure 4), especially if the effects of nutrients depend on temperature (Kendrick and Benstead, 2013; Cross et al., 2015). While a temperature scaling coefficient could standardize metabolic rates across varying temperatures (e.g., Vanni and Mcintyre, 2016), such standardization would not affect our inferences because each effect size was calculated from individuals held at the same temperature. Many of the factors that differed across studies likely drove high heterogeneity (I^2^) across effect sizes (**Table 1**), but this heterogeneity was accounted by using a mixed effects model and I^2^ was similar to that reported across other meta-analyses in ecology (Senior et al., 2016). Finally, our classification of benthic invertebrates into herbivores versus detritivores was based solely on diets fed in experiments, and may not reflect feeding ecology or the stoichiometry of feeding in the field, where animals can feed selectively on nutrient-rich biofilms (Hood et al., 2014) or forage on multiple resource types and confound trophic classification (Wolkovich et al., 2014; Snyder et al., 2015; Stoler et al., 2016). Future studies should investigate consumer feeding behavior in the field to accurately quantify bottom–up constraints on consumer growth, consumption, and excretion/egestion in green and brown benthic food webs.

## Conclusion

Our review and meta-analysis focusing on freshwater systems highlight current understanding of ecological stoichiometry in brown food webs, providing conceptual and quantitative comparison to green food webs. Although stoichiometric principles apply to both trophic systems, we suggest the nature of these principles differs in several important ways. Notably, inorganic nutrients and light availability can affect resource quantity and quality in both brown and green food webs, but in the former, both factors are likely to reduce detrital quantity via stimulated decomposition (Danger et al., 2013a; Rosemond et al., 2015) while enhancing detrital quality (Cross et al., 2003; Manning et al., 2015; Halvorson et al., 2016a), whereas in the latter, light and nutrients are likely to concurrently increase autotroph quantity while eliciting opposing effects on autotroph quality (nutrient:light hypothesis; Sterner et al., 1997). We suggest detrivorous and herbivorous consumers may respond differently to elevated resource nutrient contents, because herbivores have evolved to use resources of greater C quality and nutrient contents compared to detritivores; underlying mechanisms of these responses are also likely to differ, owing to contrasting consumption responses and assimilation efficiencies between trophic modes (Cebrian, 2004; Frost et al., 2006). Patterns in consumer-driven nutrient dynamics (CND) are also likely to differ, with egestion playing a greater relative role than excretion in brown food webs due to the recalcitrance of detrital C and nutrients, but we note excretion connects detritivores and herbivores to a shared inorganic nutrient pool, weakening direct consumer-resource feedbacks and increasing nutrient exchange between green and brown food webs (Zou et al., 2016). In a meta-analysis across controlled feeding studies, we directly compared stoichiometric constraints on invertebrates in green versus brown benthic food webs. The meta-analysis shows that herbivore and detritivore growth rates often increase with greater resource N and P contents. However, we found contrasting responses of consumption and P excretion between trophic modes, reflecting distinct herbivore and detritivore regulatory responses to elevated nutrients, probably due to contrasting resource C quality and stoichiometry.

We see several directions for continued investigation of ecological stoichiometry in both autotroph- and detrital-based systems, especially at interfaces of autotrophic and detrital-heterotrophic biomass and activity. First, there is a need for further study of how light and inorganic nutrient availability affect autotroph–heterotroph interactions on submerged detritus (Kuehn et al., 2014; Halvorson et al., 2016a) and subsequent feeding and growth of consumers (Guo et al., 2016; Stoler et al., 2016). In both trophic systems, but particularly among brown food webs, it remains difficult to accurately characterize the stoichiometry of ingested resources relative to that of bulk resources (Hood et al., 2014) and studies must address selective feeding and other foraging behavior as a mechanism of stoichiometric regulation, especially when animals may actively choose nutrient-rich resources (Dodds et al., 2014; Snyder et al., 2015; Sperfeld et al., 2017). The role of selective feeding is especially important to understand roles of multichannel consumers that can feed on both autotrophs and detritus, blurring the distinction between green and brown food webs (Wolkovich et al., 2014). Finally, our meta-analysis documents a lack of feeding experiments measuring herbivore consumption, excretion, and (especially) egestion across resource stoichiometry gradients in benthic systems. This is important because there may be distinct top–down effects of consumers on nutrient dynamics in green versus brown food webs that remain poorly known, given the lack of data. Indeed, the understudied components of CND (e.g., egestion, storage, and mortality) could notably distinguish brown food webs from their green counterparts (Atkinson et al., 2016). These directions will help workers understand the interplay of energy flow and nutrient cycling between green and brown food webs, advancing understanding of bottom–up changes like nutrient enrichment and furthering the application of ecological stoichiometry to systems along the continuum between green or brown.

## Author Contributions

ME-W led the review. HH led the meta-analysis.

## Conflict of Interest Statement

The authors declare that the research was conducted in the absence of any commercial or financial relationships that could be construed as a potential conflict of interest.
