# Supplementary material for: Comparing the Ecological Stoichiometry in Green and Brown Food Webs – A Review and Meta-analysis of Freshwater Food Webs
Source: Front Microbiol. 2017 Jun 29;8:1184. doi: 10.3389/fmicb.2017.01184 (PMC5489555; doi:10.3389/fmicb.2017.01184)
Supplement: Supplementary file 2 [file Image_1.PDF]

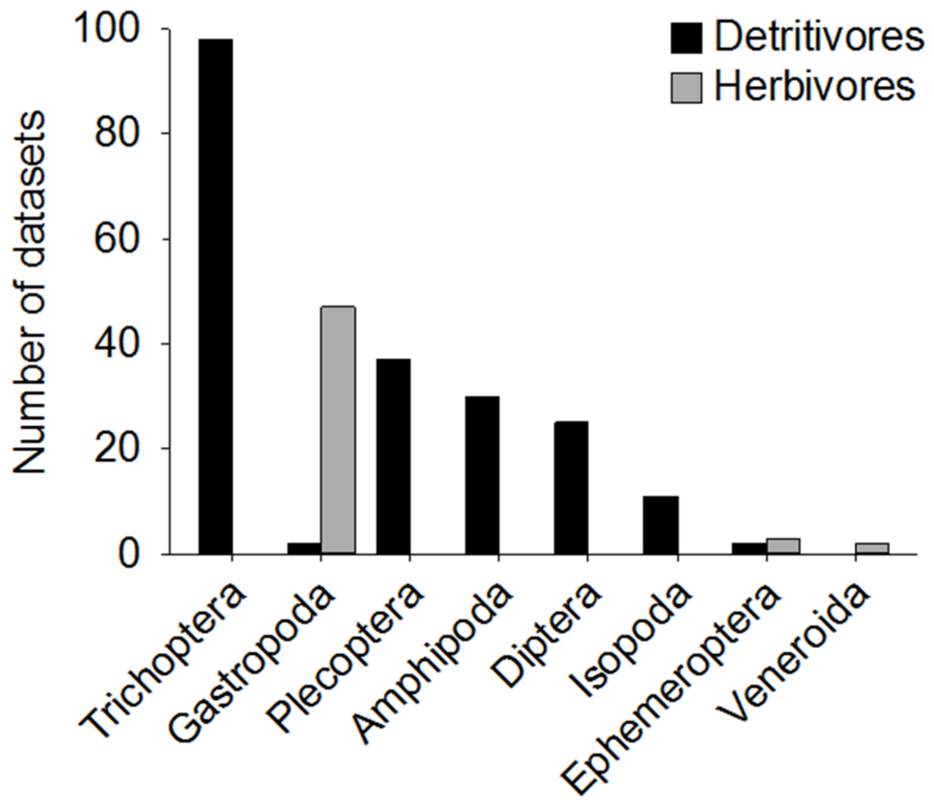

Supplemental Figure 1. Total number of datasets included in the meta-analysis of growth, consumption, excretion, and egestion responses to resource N:C and P:C contents, sorted by taxonomic Order and trophic mode.

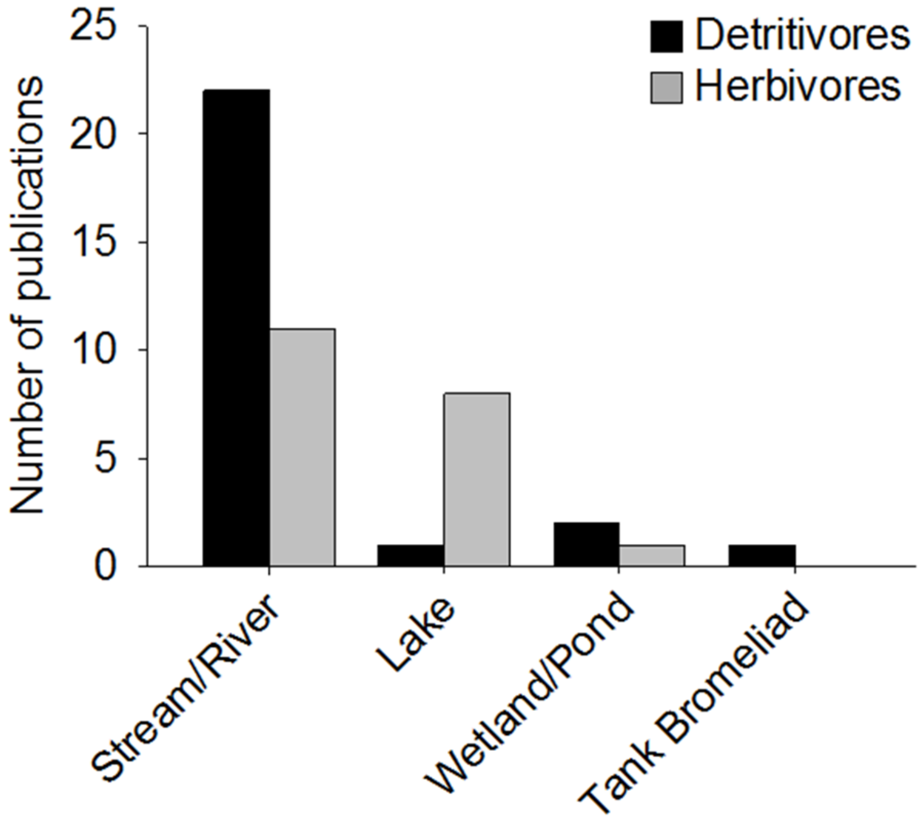

Supplemental Figure 2. Total number of publications included in the meta-analysis using benthic invertebrates from each freshwater habitat type, sorted by trophic mode.

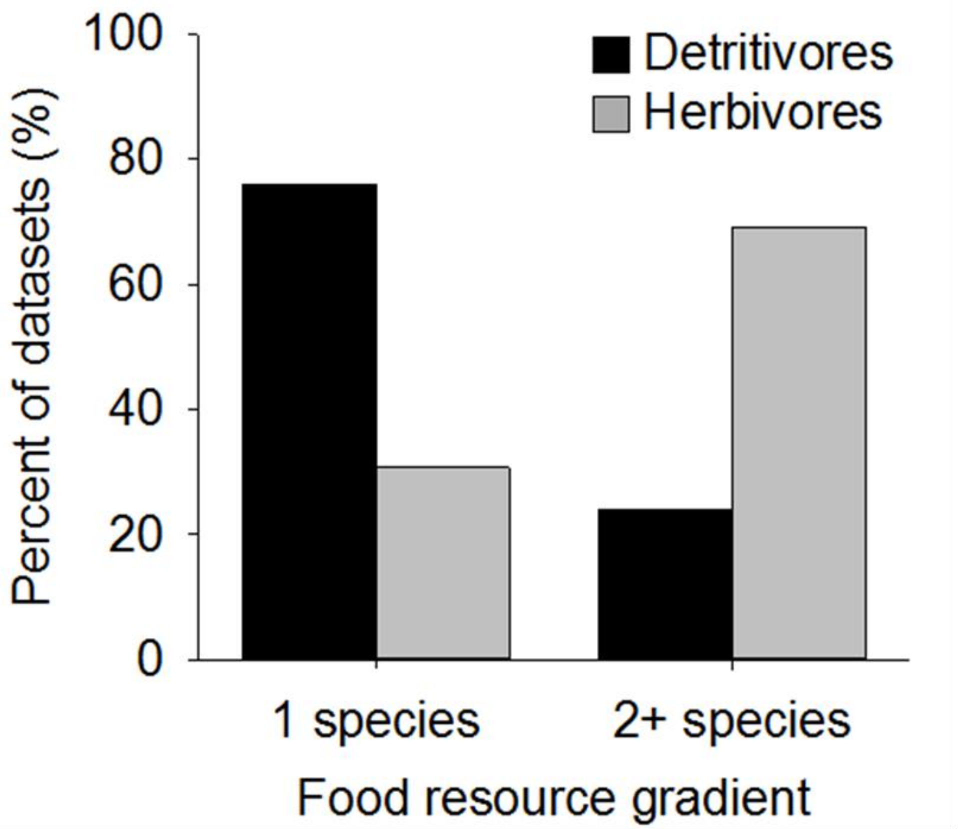

Supplemental Figure 3. Percent of datasets in the meta-analysis conducted across monospecific (1 species) or multiple species (2+ species) gradients of resource stoichiometry. Detritivore feeding studies using one single litter species were classified as monospecific.

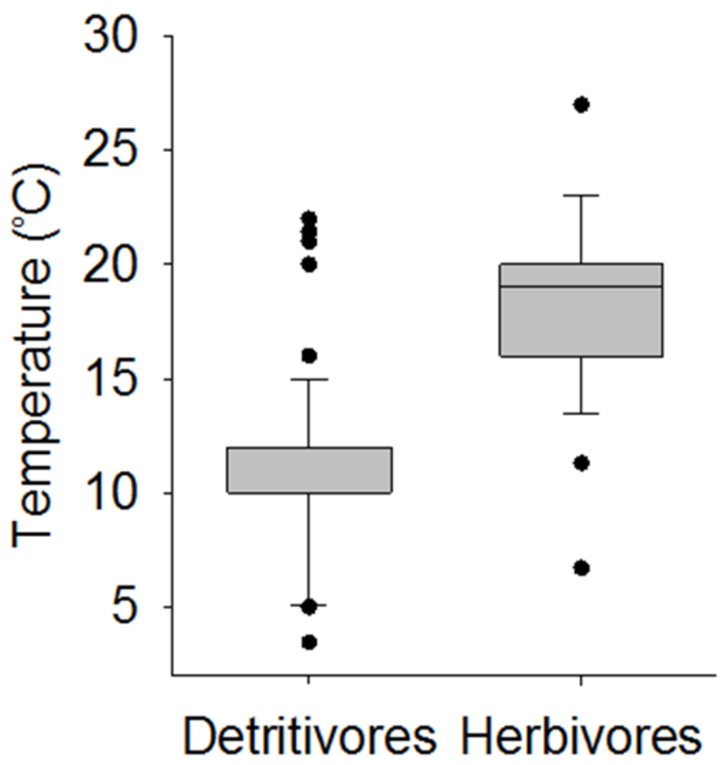

Supplemental Figure 4. Boxplot of temperatures in detritivore and herbivore feeding studies included in the meta-analysis. The black horizontal lines indicate median values within each group (the median among detritivore datasets overlaps the bottom quartile and was 10˚C).
